# Cervical cancer prevention behaviors and determinants among Indigenous women in rural Nepal

**DOI:** 10.1093/heapro/daag042

**Published:** 2026-04-11

**Authors:** Babita Bhetwal, Vinita Sharma, Anne Abbott, Ellen J Schafer

**Affiliations:** School of Public and Population Health, Boise State University, 1910 University Dr., Boise, ID 83725-1835, United States; Richard M. Fairbanks School of Public Health, Indiana University, 1050 Wishard Blvd., Indianapolis, IN 46202, United States; School of Public and Population Health, Boise State University, 1910 University Dr., Boise, ID 83725-1835, United States; School of Public and Population Health, Boise State University, 1910 University Dr., Boise, ID 83725-1835, United States

**Keywords:** cervical cancer, Tamang women, knowledge, attitude, screening

## Abstract

Cervical cancer is the leading cause of cancer-related death among Nepali women, and most have not been screened. Little is known about cervical cancer prevention behaviors [e.g. screening and human papilloma virus (HPV) vaccination] and behavioral determinants (e.g. knowledge and attitudes) among Nepal’s minority groups, and no study to date has examined Tamang women exclusively. The objective of the study was to assess cervical cancer prevention behaviors and determinants in Tamang women. We utilized an in-person, one-on-one questionnaire, administered by a research-trained Nepali woman visiting women in their homes (*n* = 250) in three municipalities of Nuwakot, Nepal. Descriptive statistics and regression models were used to characterize participants and describe factors associated with cervical cancer prevention behaviors. On average, participants were 37 years old, had a low level of knowledge about cervical cancer and preventive behaviors (18% knew at least one prevention behavior), but most had positive attitudes toward screening (76.80%) and HPV immunization (81.60%). Participants who knew what cervical cancer is (OR = 3.27, 95% CI: 1.29–8.31) or knew at least one risk factor for cervical cancer (OR = 2.51, 95% CI: 1.27–4.96) had higher odds of intending to seek screening. Study results suggest health education programs and policies need to be culturally tailored for women living in rural areas, include local stakeholders, use local communication methods, and incorporate familial support.

Contribution to Health Promotion

- Highlights the need for culturally tailored educational programs that include local stakeholders.
- Highlights the need for familial support to increase women's screening practices.
- Women from small towns have higher odds of receiving the HPV vaccine than those from rural areas, highlighting the need for better access to resources and information for the rural population.

## Introduction

Worldwide, cervical cancer is the fourth most common cause of cancer-related death, with an incidence rate of 14.12 and a mortality rate of 7.08 per 100 000 women (Wu *et al*. 2025). While the advancement of technology for early detection and vaccines against human papilloma virus (HPV, a cervical cancer risk factor) has decreased the incidence of cervical cancer in most high-income countries (Zhang *et al*. 2021), there is evidence of increasing incidence and prevalence in low- and middle-income countries (Chidebe *et al*. 2025).

Every year in Nepal, ∼2169 women are diagnosed with cervical cancer and there are 1313 associated deaths (Ferlay *et al*. 2019). Cervical cancer is the leading cause of death among Nepalese women and is most prevalent among those aged 15–44 years (Poudel and Sumi 2019). The incidence rate of cervical cancer is 16.4 per 100 000 Nepalese women, significantly higher than the World Health Organization’s (WHO’s) Cervical Cancer Elimination Initiative Goal of 4 per 100 000 women (Narasimhamurthy and Kafle 2022). The high incidence rate may be due to a lack of knowledge and awareness about cervical cancer, HPV vaccination, and cervical cancer screening (Kumari *et al*. 2022). Similarly, high mortality rates may be due to late-stage diagnosis, illiteracy, stigma related to gynecological problems among women, not letting unmarried women get gynecological examinations, misinterpretation of symptoms in 90% of cases in initial visits with healthcare providers, and lack of easy access to health care resources (Gyenwali *et al*. 2013). In addition, 80% of Nepalese women live in rural places without adequate resources, which further contribute to high mortality (Poudel and Sumi 2019).

Nepal’s government currently provides free-of-cost visual inspection with acetic acid (VIA; acetic acid is painted on tissues around the cervix and visually inspected for any changes; Rijal *et al*. 2017) for screening the early stage of cervical cancer in 64 government hospitals and health posts for women aged 30–60 years (Narasimhamurthy and Kafle 2022). VIA is a cost-effective alternative to Pap smear in low and middle-income countries (Campos *et al*. 2015). However, there is a shortage of skilled healthcare providers in rural areas (often a far distance from the 64 hospitals), a shortage of instruments and equipment for VIA examinations (Darj *et al*. 2019). Additionally, shortage of laboratories equipped to perform Pap tests, and its higher cost underscores the importance of VIA-based services (Central Bureau of Statistics 2021).

This lack of access is especially difficult for Nepal’s marginalized and Indigenous Tamang women because they are settled in geographically difficult and less accessible regions of the country, such as Nuwakot or Rasuwa. According to the 2021 Nepal Central Bureau of Statistics Report, Tamang people constitute about 5.61% of the total population of Nepal (30 666 598 people) and are the fifth largest ethnic group (Montano and Kasprzyk 2015). While cervical cancer prevention behaviors have been studied among women of Nepal, there are currently no studies of cervical cancer prevalence, screening, and HPV vaccination among Tamang women.

Thus, the aim of this study was to examine: (i) knowledge about cervical cancer, HPV, and vaccination against HPV, (ii) attitudes toward cervical cancer prevention modalities and HPV vaccination, (iii) existing practices related to cervical cancer screening and prevention, (iv) barriers and facilitators to cervical cancer prevention, (v) likelihood to participate in cervical cancer prevention behaviors or education session.

## Methods

### Study design, setting, and participants

A cross-sectional study design was employed to collect data from a convenience sample of Tamang women (*n* = 251) in the rural development committees of Nuwakot (Shivapuri, Kakani, and Likhu) through in-person interviews, between October and December 2024. Sample size was determined by reviewing a cross-sectional cervical cancer awareness study of women of reproductive age previously conducted in Likhu, Nuwakot (Bajracharya and Chalise 2021). Given that this study engaged 190 women and was able to show statistically significant findings, we aimed to interview at least 250 women.

Prior to data collection, a community health volunteer and the local government of the village assisted in disseminating study information regarding the opportunity to participate in a survey. Information spread through word-of-mouth. When data collection began, Tamang women fulfilling the eligibility criteria (i.e. those residing in Nuwakot, at least 18 years old, could provide consent, and who spoke and understood the Nepali language) were recruited. Consent was obtained verbally and did not require a signature. Those who could not provide consent or were mentally or physically unable to participate in surveys were excluded from the study.

### Theoretical or conceptual framework

Informed by the theory of planned behavior (Ajzen 1991), survey questions were built around knowledge, attitude, belief, and intention constructs. We explored HPV (knowledge), HPV vaccination (attitudes and behaviors), cervical cancer screening (knowledge, attitudes, and perceived behavioral control in terms of behavioral barriers and facilitators), and intention to seek future screening and vaccination. We used Ajzen’s (1991) definition of the following constructs:

1. Knowledge: awareness an individual has and the foundation for attitude and perception, influencing how an individual behaves.
2. Attitude: degree to which a person has a favorable or unfavorable evaluation of the behavior (Pickens 2005).
3. Intention: motivating factor that influences behavior. The stronger an individual’s intention toward a behavior, the more likely they are to perform it.
4. Perceived Behavioral Control: one’s perception of their ability (easy/difficult) to perform a behavior.

### Survey instrument and variables

The survey instrument (see English version of the survey in Supplemental Materials), which was developed and piloted in a small, similar population of Nepali women, had three parts: (i) Socio-demographic characteristics (e.g. age, age at marriage, age at first childbirth, income, household size, number of adult women at home, occupation, education, religion, help seeking, and familial cervical cancer history). Other participant characteristic variables are listed in Table 1. (ii) Knowledge, attitude, and practices of cervical cancer and prevention. (iii) Barriers and likelihood of participating in future cervical cancer prevention.

**Table 1:** Participant characteristics (*N* = 250).

Variables	Freq (%)	Mean (SD), range
Social history		
Age	–	37.49 (11.94), 18–60
Age at marriage	–	17.15 (3.48), 10–37
Age at first birth	–	18.93 (3.66), 14–36
Married	210 (84.0)	–
Has been pregnant	218 (87.2)	–
Number of pregnancies	–	2.64 (1.38), 0–7
Number of births	–	2.40 (1.26), 0–6
Small town	23 (9.2)	–
Income	–	89 788 (116 957), 5000–120 000
Education		
Secondary or higher education	61 (24.5)	–
Literate (can read and write)	137 (55%)	–
Household characteristics		
Number of dependents	–	0.94 (0.96), 0–4
Number of women	–	1.49 (0.69), 0–4
Number of girls	–	0.67 (0.86), 0–5
Household size	–	4.03 (1.81), 1–11
Employment		
Homemaker	182 (73.1)	–
Husband farmer	78 (31.2)	–
Health-related decision-making		
Husband decides for family	117 (46.8)	–
Participant decides for family	74 (29.6)	–
Husband influences participant	125 (50.0)	–
Doctor influences participant	3 (1.2)	–
Participant decides for herself	72 (28.8)	–
Hospital first if sick	92 (36.8)	–
Religion		
Hindu	12 (4.8)	–
Buddhist	235 (94.0)	–
Christian	2 (0.80)	–
Cervical cancer history		
Self	9 (3.60)	–
Sister	1 (0.40)	–
Mother	2 (0.80)	–
Mother-in-law	3 (1.2)	–

Knowledge-related questions consisted of eight closed-ended and open-ended questions to measure knowledge of cervical cancer, its risk factors, symptoms, preventive measures, and screening. A total knowledge score was calculated by summing correct responses to items related to signs and symptoms, risk factors, and prevention and early detection.

Attitudes toward cervical cancer screening and vaccination were assessed using a 5-point Likert scale (strongly disagree − strongly agree). Due to a lack of variability in some items, the attitude variables were dichotomized for analysis (1 = agree or strongly agree and 0 = any other response). An overall attitude score was calculated by summing the individual attitude variables that participants agreed to.

Cervical cancer screening and HPV vaccination practices were measured using three questions adapted from similar research conducted in Nepal (Kc *et al*. 2023): (1) Have you ever undergone screening for cervical cancer? (2) How many times have you undergone screening in the last 5 years? Based on these two questions, the interviewer marked if the participants had undergone screening in the past 5 years (regular practice); whereas, if they had undergone screening more than 5 years ago, then it was marked as irregular practice, and if they had not undergone any screening, then it was marked as no practice (Narayana *et al*. 2017). (3) Which test was performed? Pap smear/VIA/Do not know.

Barriers were assessed with an open-ended question: If you have not undergone any screening, then what are the reasons or barriers?

Intention was assessed using a 5-point Likert scale question: What is the likelihood of various prevention behaviors (i.e. undergo regular screening, vaccination against HPV, and participate in health education programs) if they are available easily? These Likert scale questions were also dichotomized for analysis.

### Data collection procedure

A master’s degree-trained (subject: law) Nepali female with certifications and training in human subjects research, the CITI Social and Behavioral Researchers 1 Basic Course, and Good Clinical Practices was trained on how to build rapport with participants, conduct the interviews, and general cervical cancer and women’s health topics. She completed the pilot test survey interviews and conducted all the paper–pencil survey interviews. Furthermore, the interviewer was introduced to potential participants by a local woman, a community health worker, who also assured participants of their confidentiality. The community health worker was sourced through local outreach and contacts and was also trained to facilitate the interviews, if necessary. Interviews were conducted one-on-one in a private place in the participant’s home and typically took 10–15 minutes to complete. Neither the study participants nor the local women were remunerated. The lead author regularly supervised the data collection process to ensure the research protocols were followed.

In each village, the community health worker introduced the interviewer to the Tamang women to build trust. When the interviewer approached the house, she greeted potential participants, explained the purpose of the visit, and gave sufficient time for the participants to think about it and ask questions if they had any. If welcomed in, she read the consent materials aloud to study participants, and consent was obtained verbally. Those who agreed to participate in the study were interviewed using a pencil-and-paper method. Since half of the participants could not write, they did not fill out the survey. Thus, the interviewer verbally interviewed each participant and used a pencil to fill out the survey on paper. At the end of the interview, an educational handout with pictures and a brief overview of cervical cancer, its symptoms, and prevention was provided. The educational handout was developed by the National Health Education Information and Communication Center and was verified for cultural relevance. The interviewer also briefly explained the content of the handout to the study participants and answered any questions they asked.

The data were collected without the participants’ identification (such as name, address, etc.), was stored securely, and was only accessible to the interviewer in Nepal and research team in the United States. When the required sample size, or number of surveys, were obtained, the interviewer made a copy of each survey and sent the originals to the lead author via FedEx. When the original documents were verified as received, the interviewer destroyed the copies she made. The data were entered into an Excel sheet, cleaned, and exported to Statistical Package for Social Science (SPSS) for analysis.

### Statistical analysis

The data were analyzed using IBM SPSS statistics version 29, and statistical significance was set at *P* < 0.05. Descriptive statistics, including frequencies, percentages, mean, standard deviation, and range were used to describe study participants. Additionally, inferential statistical analyses, including linear and logistic regression (assuming normal distribution and homoscedasticity), were used to identify statistically significant factors associated with knowledge, attitudes, and behaviors. The interviewer did not track the number of people approached versus the number of people interviewed. Thus, the response rate cannot be calculated.

## Results

Descriptive statistics for study participants (*N* = 250) are presented in Table 1. Most participants were from small rural villages (91%), married (84%), could read and write (55%), and the mean age was 37.49 years. Most of the participants (63%) reported seeking help from traditional healers (e.g. Dhami/Jhakri) prior to visiting a doctor or going to a hospital for illness. Approximately 3.6% of the participants indicated a history of cervical cancer in their immediate family.

### Knowledge about cervical cancer, HPV, and vaccination against HPV

As presented in Table 2, two-thirds of the participants knew about cervical cancer (66%), whereas less than half (46%) knew about the cervix and uterus. Participants had limited knowledge about risk factors of cervical cancer (29%). The mean overall knowledge score was 2.63, indicating that participants can answer two to three knowledge-related questions correctly out of six.

**Table 2:** Cervical cancer knowledge, attitudes, and practices (*N* = 250).

Category	Items	Frequency (%)	Mean (SD), range
Knowledge			
	Knows the cervix and uterus	115 (46.0)	–
	Heard of cervical cancer	165 (66.0)	–
	Knows a cervical cancer risk factor	72 (29.0)	–
	Knows a cervical cancer symptom	78 (31.3)	–
	Knows cervical cancer can be prevented	215 (86.0)	–
	Knows a way to prevent cervical cancer	122 (49.4)	–
	Heard of screening or Pap smear	6 (2.4)	–
	Sum of knowledge variables^a^	–	2.63 (1.52), 0–5
Attitudes^b^			
Vaccination	HPV vaccination prevents cervical cancer	204 (81.6)	–
Screening	Cervical cancer is a serious health issue	180 (72.0)	–
	Screening can find cervical cancer early	173 (69.2)	–
	Cervical cancer can affect the future	192 (76.8)	–
	All women over 30 years old should be screened	141 (56.4)	–
	Overall positive attitude sum	–	2.74 (1.24), 0–4
Practices			
	Ever undergone screening for cervical cancer	45 (18.0)	–
Number of screenings in the past 5 years	Once	30 (66.7)	–
	Twice	9 (20.00)	–
	Three times	2 (4.44)	–
	Four times	3 (6.67)	–
	More than four times	1 (2.22)	–

^a^Sum of previous six dichotomous knowledge variables; potential range = 0–6 (does not include knowledge of cervix and uterus).

^b^All attitude variables were originally measured with a 5-point Likert scale and have been dichotomized to represent positive attitudes = 1 and any other response (including do not know) = 0.

### Qualitative responses to knowledge variables

Several knowledge-related variables (e.g. knows risk factors, knows symptoms, and knows a way to prevent) were open-ended. Participants’ responses regarding risk factors included: “using husbands more/having more sex,” “a wound appears because of child birth and abortion,” and “having a swollen face.” Regarding symptoms, some participants responded: “the wound appears in the mouth, it itches, and ulcers in the mouth that protrudes,” “numbness and pain in hands and feet,” and “discharge of white fluids.” Responses regarding prevention measures included: “don’t have many kids” and “uterine catheterization.”

### Attitude toward cervical cancer prevention (vaccination and screening)

As shown in Table 2, most participants (82%) believed that HPV vaccination prevents cervical cancer. Regarding cervical cancer screening, 72% of participants identified cervical cancer as a serious health issue, and 69% believed screening could detect cervical cancer early. The mean attitude score is 2.74, indicating positive leaning attitudes toward two to three of five attitude variables used to create the score.

### Practices related to cervical cancer prevention

Only 18% of the study participants had undergone screening for cervical cancer, and of those, 66.7% underwent screening at least once in the last 5 years. Only one participant was able to name the type of test performed.

### Barriers and likelihood of participating in cervical cancer prevention

Variations of top five most common reasons for not practicing cervical cancer prevention behaviors were: “Nothing had happened.” (53.36%); “I did not get sick.” (22.79%); “I have no symptoms.” (11.91%); “I would go after getting sick.” (4.66%); “I do not know about prevention.” (2.59%). However, most participants (96%) indicated an intention to seek cervical cancer screening, were willing to have HPV vaccination (93%), and were likely to participate in programs to increase knowledge (93%).

### Factors associated with cervical cancer knowledge

Table 3 demonstrates the factors associated with each of the outcome variables. In bivariate analysis, several factors were significantly associated with knowledge, including having more than a basic level of education, being older, the ability to read and write, making health decisions for the family, and making their own health decisions (i.e. makes decisions independently for one’s self and someone else is not making decisions for them). When all the factors were combined into one model, the following factors remained significantly associated with knowledge: the ability to read and write (*b* = 1.1) and participants making their own health decisions (*b* = −0.42). Only the statistical analysis from the final model is presented in the table; the # sign is used to indicate factors independently associated with knowledge but not significant in the full model.

**Table 3:** Factors associated with and predictive of cervical cancer knowledge, attitudes, and behaviors (*N* = 250).

Predictor variables	Cervical cancer knowledge (b, SE)	Positive screening attitude (b, SE)	Positive HPV vaccination attitude (OR, 95% CI)	Likeliness of screening (OR, 95% CI)	Likeliness of HPV vaccination (OR, 95% CI)
Read and write	1.13 (0.18)***	#	#	–	–
More than basic education	–	–	2.72 (1.01, 7.33)*	–	–
Age	#	–	–	–	–
Participant decides for self	−0.42 (0.20)*	–	–	–	–
Participant decides for family	#	–	–	–	–
Husband farmer	–	−0.34 (0.17)*	–	–	–
Read and write	–	#	–	–	–
Has screened	–	#	–	–	–
Knowledge sum	–	0.24 (0.05)***	–	–	–
Knows cervical cancer can be prevented	–	–	2.34 (1.05, 5.22)*	–	–
Knows cervix and uterus	–	–	–	#	–
Knows what cervical cancer is	–	–	–	3.27 (1.29, 8.31)*	–
Knows a risk factors	–	–	–	2.51 (1.27, 4.96)**	–
Knows a symptoms	–	–	–	#	–
Knows a way to prevent cervical cancer	–	–	2.34 (1.05, 5.22)*	–	–
Positive attitude: cervical cancer is a serious health issue	–	–	–	–	#
Positive attitude: screening can find cervical cancer early	–	–	–	–	#
Positive attitude: HPV vaccination can prevent cervical cancer	–	–	–	–	2.41 (1.09, 5.33)*
Agree, screening is uncomfortable	–	–	–	–	2.16 (1.22, 3.85)**
Small town	–	–	–	–	3.46 (1.29, 9.27)*

^*^ *P* < 0.05, ** *P* < 0.01, *** *P* < 0.001; # significant in bivariate analysis but not in full model; – not significantly associated with the outcome variable.

### Factors associated with positive screening and vaccination attitudes

Bivariate analysis was run for each independent variable listed in Table 2. However, only the variables that were significantly associated with one’s attitude toward screening were included in additional models (i.e. ability to read and write, husband is a farmer, has previously been screened, and overall knowledge sum score). In the final model, participants whose husbands were farmers tended to have a less positive attitude toward screening (*b*  *=* −0.34). Also, the knowledge sum variable was significantly associated with a more positive attitude toward screening (*b* = 0.24).

In bivariate analysis, the following factors were significantly associated with vaccination attitudes: more than basic education, ability to read and write, knowledge that cervical cancer can be prevented, and overall knowledge sum score. In the final model, participants reporting that they had more than a basic education had higher odds of having greater cervical cancer knowledge compared with those with only a basic education or no education (OR = 2.72; CI = 1.10–7.33). Similarly, those who reported knowing that cervical cancer can be prevented had higher odds of having greater cervical cancer knowledge compared with those who did not have this awareness (OR = 2.34; CI = 1.05–5.22).

### Factors significantly associated with cervical cancer screening and vaccination

In bivariate analysis, the following factors were significantly associated with the likelihood of screening: knows what cervix and uterus are, knows what cervical cancer is, knows at least one cervical cancer risk factor, knows cervical cancer symptoms, knows a way to prevent cervical cancer, and overall knowledge sum score. In the final model, the following factors remained significant: knows at least one risk factor (OR = 2.51, CI = 1.27–4.96) and knows what cervical cancer is (OR = 3.27, CI = 1.29–8.31).

In bivariate analysis, the following factors were significantly associated with the likelihood of HPV vaccination: attitude regarding cervical cancer as a serious health issue, attitude regarding cervical cancer screening (can find cancer early), attitude that HPV vaccination can prevent cervical cancer, agree screening is uncomfortable, and from a small town. When all of the factors were combined into one model, the following factors remained significant with likelihood of HPV vaccination: a positive attitude toward HPV vaccination preventing cervical cancer (OR = 2.41, CI = 1.09–5.33), agreeing that cervical cancer screening is uncomfortable (OR = 2.16, CI = 1.22–3.85), and living in a small town (OR = 3.46, CI = 1.29–9.27).

## Discussion

This study assessed Tamang women’s knowledge, attitudes, and behaviors related to cervical cancer and prevention.

### Knowledge about cervical cancer, screening, and prevention

The participants had low overall knowledge and neutral to positive attitudes toward cervical cancer prevention behaviors. Previous studies have included Nepali women but have not explored cervical cancer and prevention among Tamang women exclusively. Our findings suggest cervical cancer knowledge was low in our study population, which is consistent with other studies conducted among 180 women aged 30–60 years old residing in Urgachandi Nala VDC (Acharya Pandey and Karmacharya 2017); and among 729 women aged 30–60 years residing in the semi-urban area of Pokhara Metropolitan City of Nepal (Shrestha *et al*. 2022b). Most participants knew about cervical cancer, whereas less than half knew about the cervix and uterus (i.e. anatomical location and function). This knowledge gap suggests participants may have limited health literacy about cervical cancer and anatomy. Thus, to strengthen the current cervical cancer prevention efforts, future campaigns and programs should focus on basic education on reproductive anatomy and terminology. For instance, as a result of an educational intervention that included three sessions of video presentation, PowerPoint presentation, and discussions in Tangali, Bangladesh, participants gained a significant increase in knowledge and perception about cervical cancer and its prevention (Mili *et al*. 2025).

Interestingly, participants who indicated making their own health decisions tended to have lower cervical cancer knowledge. Given the cultural relevance of multigenerational households and a patriarchal society in Nepal (Nepal *et al*. 2023), it is possible that women making their own decisions may not have family support. While they might have the autonomy to make decisions for themselves, their knowledge or support to enact screening or prevention behaviors may be limited if there is no one else in the family or society to consult about health decisions. Public health programs must consider women who are making health decisions alone as a potentially vulnerable group. Future interventions could incorporate community-based outreach and peer education models, such as Nepal’s recently launched HPV immunization program (Pandav *et al*. 2025).

### Attitude toward cervical cancer prevention behaviors

Participants indicated positive attitudes toward cervical cancer prevention. Several studies conducted regarding awareness and attitudes about cervical cancer among Nepalese women have found positive attitudes toward cervical cancer screening among ethnic minority women, as compared with Brahmin and Chhetri (upper caste) (Johnson *et al*. 2014, Thapa *et al*. 2018, Kumari *et al*. 2022). However, in our study, participants whose husbands were farmers had significantly lower or less positive screening attitudes. Interestingly, household income was not significantly associated with screening attitudes in our model, indicating women from farming communities face unique barriers such as limited health literacy, time constraints, access to resources, or support from partners (Adjei Boakye *et al*. 2018). A study conducted among Indonesian women has shown the significant positive impact of a husband’s support in relation to participation in cervical cancer screening among women (Juwitasari *et al*. 2021). Additionally, most of the participants said that their husband influences their health-related decisions. Thus, if a farmer-husband does not have accurate knowledge (or access to knowledge/resources) about the importance of cervical cancer prevention, women may be less likely to undergo screening. Therefore, future research and public health practice may benefit from exploring strategies to engage men in reproductive health education and to help foster an environment where informed decisions are supported within the household.

The odds of participants having positive attitudes toward HPV vaccination were significantly higher among those with more than a basic education. These findings are not surprising, given previous HPV-related studies with comparable results highlighting the importance of education (Adjei Boakye *et al*. 2018, Leung and Law 2018, Castañeda-Avila *et al*. 2022). Increasing education and literacy rates among women and girls in rural Nepali villages may work to empower them and prevent cervical cancer and other diseases. Nepali officials might consider implementing interventions similar to other middle- and low-income countries. For example, Ghana implemented a multifaceted educational interventional program about cervical cancer among higher secondary level school students to increase cervical cancer prevention knowledge at an early stage, which translated to positive behaviors and attitudes later (Dedey *et al*. 2024). After the implementation of this program, cervical cancer knowledge significantly improved among participants from 79.1% to 92.3%.

### Cervical cancer prevention behavior and determinants

Very few of the participants (18%) were “ever” screened for cervical cancer. Most participants who had “ever” been screened for cervical cancer reported they had been screened because a health camp was organized in the village, or they had some symptoms, such as pain and burning, prompting them to seek screening. Often, the health camps in Nepal offer exams for uterine prolapse, a common issue among Nepali women (Subedi 2010). Many women may have had a pelvic exam at a health camp to check for uterine prolapse, but if not reporting symptoms related to cervical cancer, it is unlikely that cervical cancer screening happened as well. Thus, the reported prevalence of cervical cancer screening may be an overrepresentation of the true screening rate, and more research into how often cervical cancer screening is conducted in health camps is warranted. This potential misunderstanding could reflect a broader gap in health literacy and reproductive health knowledge seen throughout the study.

Most of the participants did not know that HPV vaccination exists or that it prevents cervical cancer. Participants would often ask the interviewer for more information and she would explain that the vaccine would prevent HPV, which can help prevent cervical cancer. Thus, toward the end of the survey, when asked about their likelihood to get vaccinated, the responses were more positive. Recently, Nepal launched a national vaccination program to vaccinate 1.6 million adolescent girls and intends to include HPV vaccination in the national immunization schedule (Neupane *et al*. 2025). Given our findings of low knowledge regarding HPV and vaccination, the implementation of this campaign could help to improve knowledge and lead to an important prevention behavior for women and girls in the country moving forward.

Considering this, it is worth revisiting that study participants living in small towns within the rural areas had more than three times the likelihood of getting the vaccine compared with women living in smaller, more rural areas, suggesting women from small towns had awareness about vaccines, likely stemming from better access to healthcare resources. Other contributing factors may be socioeconomic status, education, and accessibility (Basu 2019). Financial and healthcare resource constraints can influence attitudes toward vaccination (Ye and Su 2025), and women in small towns may have had both the knowledge and financial means to afford the vaccine. Similarly, those who had more than a basic education, as well as those who reported knowing that cervical cancer can be prevented, had more than double the odds of having positive attitudes toward HPV vaccination. Additionally, study participants who believed HPV vaccination could prevent cervical cancer and those who agreed that cervical cancer screening is uncomfortable both had more than double the odds of getting the vaccine compared with women without those beliefs.

All these findings shed light on what may be useful focus areas for future vaccine programs in Nepal and may be especially useful in ensuring they are inclusive of ethnic minorities like Tamang women. One specific area where more research is needed is the relationship between discomfort with screening and the likelihood of vaccination. While public health practitioners should support both primary and secondary prevention behaviors, it is possible that participants were likely to be vaccinated because they did not want to go through the discomfort of screening. If so, this finding may have implications for cervical cancer prevention education and communication.

### Implications and recommendations

The findings of this study show a knowledge gap and low participation in cervical cancer prevention behaviors. Additionally, more than half of the participants were unable to describe what a cervix is, indicating low reproductive health literacy, which can contribute to low screening rates, delayed healthcare-seeking behavior, and misconceptions about cervical cancer and its prevention (Lindau *et al*. 2002). These delays result in later-stage cancer diagnoses and increased morbidity (Amalraj *et al*. 2009). Therefore, to bridge this gap, emphasis should be given to reproductive health education at schools and female community health workers should be trained to visit door-to-door to provide information. Cultural tailoring of health education is essential to effectively reach the Tamang women. Peer health educators from the Tamang community may help normalize the discussion about reproductive health in a culturally sensitive way. Furthermore, communication of information through the radio may be a useful approach to educate people about disease prevention, particularly in rural areas (Saha *et al*. 2021), especially since some participants in our study reported learning about cervical cancer on the radio. Radio programs about cervical cancer and reproductive health in the Tamang language could be another way to bridge the knowledge gap.

Most of the Tamang women we interviewed tended to seek help from traditional healers when they get sick, before considering other healthcare options. Including traditional healers as stakeholders in designing an education program may be effective, as women already trust and seek help from them. Therefore, culturally tailored interventions could include Tamang peer health educators, radio programs about sexual/reproductive health in the Tamang language, and engaging traditional healers in educational programs.

While the screening rate may have been overestimated in our sample, screening was positively associated with knowledge variables. Therefore, emphasis should be given to educational programs and the provision of screening services (Campos *et al*. 2017). VIA screening services are available free-of-cost at most of the government health posts or hospitals (Shrestha *et al*. 2022b), but our sample did not have any knowledge about VIA and did not know that it is recommended to be screened once every 5 years after the age of 30 (Dangal *et al*. 2024). The cost of a Pap smear test is about 5000–10 000 Nepali rupees ($3–6USD) (Shrestha *et al*. 2022a), which is some of our study participants’ annual income, particularly those of farming households. Therefore, to overcome these barriers, the government should consider policies and programs at the national level to educate women about screening guidelines, available screening/vaccination services, and their associated costs.

### Strengths and limitations

As far as the authors are aware, this is the first study conducted to assess cervical cancer prevention-related knowledge, attitudes, and practices among Tamang women of Nepal. It was a community-based study, and its findings have direct implications for designing culturally appropriate health education interventions.

Although efforts were made to avoid biases, the study has some limitations. Social desirability bias may have existed during interviews, as the topic was something that women do not want to talk to anyone about. In retrospect, some of the attitude-related questions that we asked were better at measuring knowledge rather than attitudes. This led to a limitation in our ability to fully capture attitude as originally intended. Additionally, the attitude variables were measured on a Likert scale, which seems inappropriate for these rural population, who have low health literacy and numeracy (Chachamovich *et al*. 2009, Flaskerud 2012).

Lastly, given the convenience sampling methods, the results of this study may not be representative or transferable to all Tamang women of Nepal.

### Conclusion

This study assessed cervical cancer prevention behaviors among Tamang women using open and closed-ended questions, which allowed participants to reflect on what they knew. The findings indicate that while participants were shy, many were open to discussing cervical cancer, even though it is often considered a taboo topic and cultural norms discourage conversation about reproductive and sexual health. Additionally, cervical cancer knowledge and screening practices were low; however, participants were likely to participate in education or prevention activities if they were easily accessible. Therefore, future research and practice should focus on providing accessible, low barrier, and low-cost education, immunization, and screening services to rural populations to bridge the gap in knowledge among rural and ethnic minority women.

## Supplementary Material

daag042_Supplementary_Data

## Data Availability

The data used in this study is available upon request to the main author.
