# Supplementary material for: Cervical cancer prevention behaviors and determinants among Indigenous women in rural Nepal
Source: Health Promot Int. 2026 Apr 11;41(2):daag042. doi: 10.1093/heapro/daag042 (PMC13070706; doi:10.1093/heapro/daag042)
Supplement: daag042_Supplementary_Data [file daag042_supplementary_data.pdf]

अन्तरवार्ता संख्या/Interview ID __________ मति/Date________________

घरधुरी संख्या/Household ID________________ समय/Time________________

**Introduction to Survey/सर्वेक्षणको परिचय**

Hi,

My name is _____________________, I am a research volunteer, and our team is conducting a survey in some of the villages of Nuwakot about cervical cancer and behavior toward its prevention among Tamang women. The questions usually take about 10-20 minutes. All the answers you give will be confidential and will not be shared with anyone other than members of our research team. You don't have to be in the survey, but we hope you will agree to answer the questions since your views are important. If I ask you any question you don't want to answer, just let me know and I will go on to the next question or you can stop the interview at any time.

Do you have any questions?

May I begin the interview now?

Signature of interviewer______________________ Date___________________________

**नमास्कार,**

**मेरो नाम _________________ हो, म एक अनुसन्धान स्वयंसेवक हुँ र हाम्रो टोलीले नुवाकोटका केही गाउहँरूमा पाठेघरको मुखको क्यान्सर र तामाङ महलिाहरूमा यसको रोकथाम गर्ने व्यवहारको बारेमा सर्वेक्षण गररिहेको छ।**

**प्रश्नहरू सामान्यतया 10-20 मनिट लाग्छ। तपाईंले दनिुहुने सबै जवाफहरू गोप्य हुनेछन् र हाम्रो अनुसन्धान टोलीका**

अन्तरवार्ता संख्या/Interview ID __________ मिति/Date________________

घरधुरी संख्या/Household ID________________ समय/Time________________

सदस्यहरू बाहेक अरू कसैसगँ साझा गरनि छैन। तपाईंले सर्वेक्षणमा भाग लिनु अनविार्य छैन, तर हामी

आशा गर्दछौं कि

तपाईं प्रश्नहरूको जवाफ दनि सहमत हुनुहुनेछ कनिभने तपाईंका वचिारहरू यस अनुसन्धानको लागि

महत्त्वपूर्ण हुनेछन्।

यदि मैले तपाईलाई कुनै प्रश्न सोधें र तपाईले जवाफ दनि चाहनुहुन्न भने, मलाई थाहा दिनुहोस् र म

अर्को प्रश्नमा जान्छु

वा तपाईले कुनै पनि समय अन्तर्वार्ता रोक्न सक्नुहुन्छ।

तपाईँको केही प्रश्न छन्?

के म अन्तर्वार्ता सुरु गर्न सक्छु?

अन्तर्वार्ताकर्ताको हस्ताक्षर_______________________ मिति____________________________

## SURVEY INSTRUMENT

| | |
|---|---|
| 1. | What is your age? _________________________ (# of years)<br>तपाईँको उमेर कति हो? _______________________ (#वर्षमा) |

## CERVICAL CANCER QUESTION BY THEMES / शिर्षक बमोजिम पाठेघरको मुखको क्यान्सर सम्बन्धि प्रश्नहरु

| KNOWLEDGE /ज्ञान |
|---|

अन्तरवार्ता संख्या/Interview ID _________ मतिि/Date_______________

घरधुरि संख्या/Household ID_______________ समय/Time_______________

| | |
|---|---|
| A. | **Anatomy (एनाटोमी)** |
| 2. | Do you know what the uterus and cervix are? / तपाईँलाई पाठेघर र पाठेघरको मुख के हो थाहा छ? |
| | ☐ Yes / छ<br>☐ No / छैन<br>☐ Do not know/ थाहा छैन<br>☐ Prefer not to answer/ उत्तर दनि रुचाउदँनि |
| B. | **Cervical Cancer / पाठेघरको मुखको क्यान्सर** |
| 3. | Have you ever heard of cervical cancer? / के तपाईलँे कहलि्यै पाठेघरको मुखको क्यान्सर बारे सुन्नु भएको छ.? |
| | ☐ Yes /छ<br>☐ No/छैन<br>☐ Do not know / थाहा छैन<br>☐ Prefer to answer//उत्तर दनि रुचाउदँनि |
| | **Risk factors are those elements that increase the chance of disease. जोखमिका कारणहरु ती तत्वहरु हुन् जस्ले रोग लाग्ने सम्भावना बढाउछ।** |
| 4. | Please share with me risk factors of cervical cancer if you know any. / तपाईलँे जान्नु भएको कुनै पाठेघरको मुखको क्यान्सरका जोखमिका कारण हरु मलाई बताईदनिुहोसन्। |
| | |
| C. | Signs and Symptoms /लक्षणहरु<br>**Symptoms are the changes observed in the body after getting disease. / रोगका लक्षणहरु भन्नाले रोग लागेपछि शिररिमा देखनि नकारातमक परविर्तनहरु हुन्।** |
| 5. | Please share with me the signs and symptoms of cervical cancer if you know any. / तपाईलँे जान्नु भएको कुनै पाठेघरको मुखको क्यान्सरका लक्षणहरु मलाई बताईदनिुहोसन्। |
| | |

अन्तरवार्ता संख्या/Interview ID _________ मति/Date______________

घरधुरी संख्या/Household ID______________ समय/Time______________

| | | |
|---|---|---|
| | | |
| D. | **Prevention and Early Detection/ रोकथाम र प्रारम्भकि चरणमा पहचिान** | |
| 6. | Do you think cervical cancer can be prevented? / के तपाईँलाई पाठेघरको मुखको क्यान्सरको रोकथाम गर्न सकन्छि जस्तो लाग्छ? | |
| | ☐ **Yes/ लाग्छ**<br>☐ **No/लाग्दैन** | ☐ **Do not know/ थाहा छैन**<br>☐ **Prefer not to answer/उत्तर दनि रुचाउदँनि** |
| 7. | **Please share with me some of the ways to prevent cervical cancer if you know any. / तपाईंले जान्नु भएको कुनै पाठेघरको मुखको क्यान्सरका रोकथामका उपायहरु मलाई बताईदनिुहोसन्।** | |
| | | |
| 8. | Have you ever heard about pap smear tests or visual inspection with acetic acid (VIA)? / के तपाईँले प्याप स्मयिर परक्षिण र एसटिकि एसडि बाट दृस्यात्मक रुपमा परक्षिण (VIA) गर्ने बारे सुन्नुभएको छ? | |
| | ☐ **Yes /छ**<br>☐ **No/छैन** | ☐ **Do not know/थाहा छैन**<br>☐ **Prefer not to answer/उत्तर दनि रुचाउदँनि** |
| **Skip question 9 if the answer to question 8 is other than “Yes”. / यदि प्रश्न 8 को उत्तर “छ” बाहेक हो भने प्रश्न 9 सोध्न पर्दैन।** | | |
| 9. | **If yes, do you know the purpose of the Pap smear screening test? / यदि छ भने, के तपाईँलाई प्याप स्मयिर स्क्रनिङ्गि (pap smear) परक्षिण को प्रयोजन थाहा छ?** | |
| | ☐ **Yes/छ** | ☐ **Do not know/ थाहा छैन** |

| | | |
|---|---|---|
| | ☐No/छैन | ☐Prefer not to answer/उत्तर दिन रुचाउदैँन |

| **PRACTICES** | |
|---|---|
| Now I'm going to ask you about tests a healthcare worker can do to check for cervical cancer, which is cancer in the cervix. The cervix connects the womb to the vagina. To be checked for cervical cancer, a woman is asked to lie on her back with her legs apart. Then the healthcare worker will use a brush or swab to collect a sample from inside her. The sample is sent to a laboratory for testing. This test is called a Pap smear or HPV test. Another method is called a VIA or Visual Inspection with Acetic Acid. In this test, the healthcare worker puts vinegar on the cervix to see if there is a reaction. **अब म तपाईँलाई सवास्थकर्मिले गर्न सक्ने पाठेघरको मुखको क्यान्सको परक्षिणको बारेमा सोध्छु। पाठेघरको मुखले पाठेघलाई यौनसिणग जोड्दछ। पाठेघरको मुखको क्यानस्रको परक्षिणको लागि माहलिालाई खुट्टा फट्टाएर उत्तानो परेर सुत्न अनुरोध गरन्छि। त्यसपछि स्वास्थकर्मीले ब्रस वा स्वाबको सहयोग बाट उनी भित्र बाट नमुना संकलन गर्छन। नमुनालाई प्रयोगशालामा परक्षिणको लागि पठाईन्छ। यस परक्षिणलाई प्याप स्मयिर वा** HPV **परक्णि भनन्निछ। अर्को परक्षिणको तरकिालाई** VIA **अथवा एसटिकि एसडिको सहयोगले दृस्यात्मक रुपमा गरनि परक्षिण भनन्छि। यस परक्षिणमा स्वास्थकर्मीले पाठेघरको मुखमा अमलिो पदार्थ हालेर यद कुिनै प्रतक्रिीया गरेको छ वा छैन भनेर हेर्नेछन्।** | |
| 10. | Have you ever undergone screening for cervical cancer? / के तपाईँले कहलि्यै पाठेघरको मुखको क्यान्सरको लागि परिक्षिण गराउनु भएको छ? |
| | ☐**Yes/ छ**<br>☐**No/छैन**<br>☐**Do not know/ थाहा छैन**<br>☐**Prefer not to answer/उत्तर दनि रुचाउदैँनि** |
| **If answer to question 10 is "No", skip questions 11 and 12. /यद प्रिश्न 10 को उत्तर "छैन" हो भने प्रश्न 11 र 12 सोध्न पर्दैन।** | |
| 11. | How many times have you undergone screening in the last 5 years? / तपाईँले पछलि्लो ५ बर्षमा कति पटक परक्षिण गराउनुभएको छ? |
| | ☐**One time/ एक पटक**<br>☐**Two time /दुई पटक** |

| | |
|---|---|
| | ☐ **Three time / तीन पटक**<br>☐ **Four time / चार पटक**<br>☐ **More than Four time/ चार पटक भन्दा धेरै** |
| 12. | Which test was performed? / कुन परिक्षण गराईएको थियो? |
| | ☐ PAP Smear/प्याप स्मयिर<br>☐ VIA /VIA<br>☐ **Do not know / थाहा छैन** |
| **Skip question 13 if the answer to question 10 is "Yes". / यदि प्रश्न 10 को उत्तर "हो" भने प्रश्न 13 सोध्न पर्दैन।** | |
| 13. | If you had not undergone any screening, then what are the reasons for it? / यदि तपाईँले परिक्षण गराउनुभएको छैन् भने, त्यसको कारण के थियो? |
| | |
| 14. | What is the likelihood of up taking the following preventative modalities if they are easily available and accessible? / यदि निम्नि रोकथामका उपयाहरु सहज रुपमा पहुँचयोग्य छ भने यसको प्रयोग गर्ने सम्भाव्यता कति छ? |

| | Preventative modalities रोकथामका उपायहरु | VERY UNLIKELY/ एकदमै सम्भव छैन | UNLIKELY/ सम्भव छैन | LIKELY/सम्भव छ | VERY LIKELY/एकदमै सम्भव छ | ALMOST CERTAIN/ लगभग निश्चित |
|---|---|---|---|---|---|---|
| a. | Cervical cancer screening / | ☐ | ☐ | ☐ | ☐ | ☐ |

**अन्तरवार्ता संख्या/Interview ID __________** **मिति/Date________________**

**घरधुरी संख्या/Household ID________________** **समय/Time________________**

| | | | | | | |
|---|---|---|---|---|---|---|
| | पाठेघरको मुखको क्यान्सरको परिक्षिण | | | | | |
| b. | HPV vaccination/ HPV वृद्धिको खोप | ☐ | ☐ | ☐ | ☐ | ☐ |
| c. | Programs to enhance knowledge / ज्ञान बृद्दि गर्ने कार्यक्रमहरु | ☐ | ☐ | ☐ | ☐ | ☐ |

| **ATTITUDES (मनोवृत्ती)** | | | | | | | | |
|---|---|---|---|---|---|---|---|---|
| | **STATEMENTS/ कथनहरु** | **1 STRONGLY DISAGREE/ पूरण रुपमा असहमत** | **2 DISAGREE/ असहमत** | **3 NEUTRAL/ तटस्थ** | **4 AGREE/ सहमत** | **5 STRONGLY AGREE/ पूरण रुपमा सहमत** | **DO NOT KNOW/ थाहा छैन** | **PREFER NOT TO ANSWER/ उत्तर दिनि रुचाउदैंनि** |
| 15. | **Cervical cancer is a serious health issue. / पाठेघरको मुखको क्यान्सर एक जटलि स्वास्थ समस्या हो।** | ☐ | ☐ | ☐ | ☐ | ☐ | ☐ | ☐ |
| 16. | **Cervical cancer screening such as pap smear and VIA can early detect cervical cancer. /प्याप स्मयिर र VIA जस्ता पाठेघरको मुखको क्यान्सर परिक्षिणले प्रारम्भकि चरणमा पाठेघरको मुखको क्यान्सरको पहचािन गर्न** | ☐ | ☐ | ☐ | ☐ | ☐ | ☐ | ☐ |

अन्तरवार्ता संख्या/Interview ID __________                    मिति/Date________________

घरधुरि संख्या/Household ID________________                    समय/Time________________

| | | | | | | | | |
|---|---|---|---|---|---|---|---|---|
| | सक्छ। | | | | | | | |
| 17. | All women above 30 years must be screened for cervical cancer. / ३० बर्ष माथिका सबै महिलाले पाठेघरको मुखको क्यानसरको लागि परिक्षण गराउनुपर्छ। | ☐ | ☐ | ☐ | ☐ | ☐ | ☐ | ☐ |
| 18. | Cervical cancer can affect you in the future. / पाठेघरको मुखको क्यान्सरले तपाईललाई भविष्यिमा असर गर्न सक्छ। | ☐ | ☐ | ☐ | ☐ | ☐ | ☐ | ☐ |
| 19. | Vaccination against HPV prevents cervical cancer. / HPV बिरुध्दको खोपले पाठेघरको मुखको क्यान्सरको रोकथाम गर्छ। | ☐ | ☐ | ☐ | ☐ | ☐ | ☐ | ☐ |
| 20. | Cervical cancer screening is uncomfortable. / पाठेघरको मुखको क्यान्सरको परकि्षण असहज हुन्छ। | ☐ | ☐ | ☐ | ☐ | ☐ | ☐ | ☐ |
| 21. | Womens who are old or had hysterectomy do not need to get screened. बृध्द महिला वा पाठेघर निकालेको महिलाले | ☐ | ☐ | ☐ | ☐ | ☐ | ☐ | ☐ |

अन्तरवार्ता संख्या/Interview ID _________ मतिि/Date_______________

घरधुरि संख्या/Household ID_______________ समय/Time_______________

| | | | | | | | | |
|---|---|---|---|---|---|---|---|---|
| | परकि्षण गराउनुपर्दैन। | | | | | | | |
| 22. | Every woman may not get cervical cancer. / सबै महलिालाई पाठेघरको मुखको क्यान्सर हुदैंन। | ☐ | ☐ | ☐ | ☐ | ☐ | ☐ | ☐ |

**DEMOGRAPHICS INFORMATION/ जनसंख्यि जानकारी**

| | | |
|---|---|---|
| 23. | How many people live in your home? / तपाईँको घरमा कति जिना बस्नुहुन्छ? | ____________________ |

| | | | |
|---|---|---|---|
| 24. | What is your marital status? तपाईँको बैवाहकि अवश्था के हो? | | |
| ☐Never married/ कहलि्यै बविाह नगरेको | ☐Ever had sex/यौन सम्पर्क गरेको<br>☐Never had sex/ कहलि्यै यौन सम्पर्क नगरेको | ☐Widowed/ वधिवा | ☐Never remarried / कहलि्यै पुनर्बविाह नगरेको<br>☐Remarried/ पुर्नववािह गरेको |
| ☐Married/living together /बविाहति / सगैं बस्ने | | ☐Divorced/Separated (परपाचुके भएको/ छुट्टएिको) | |

| | | | |
|---|---|---|---|
| 25. | At which age did you get married? /तपाईँको बविाह कति वर्षको उमेरमा भएको थयिो? | _______ (#of years/वर्षमा) | ☐Don't apply/ लागु हुदैंन |
| 26. | What was your age when you gave birth to your first child? /तपाईंले आफ्नो पहलिो सन्तान जन्म दर्दिां कति उमेरको हुनुहुन्थ्यो? | _______ (#of years/वर्षमा) | ☐Don't apply/ लागु हुदैंन |
| 27. | How many times did you get pregnant? /तपाईँ कति पिटक गर्भवती हुनु भएको छ? | __________ | ☐Don't apply/ लागु हुदैंन |

अन्तरवार्ता संख्या/Interview ID __________ मिति/Date________________

घरधुरि संख्या/Household ID________________ समय/Time________________

| | | | |
|---|---|---|---|
| 28. | How many live births did you have? / तपाईंले कति पटक जीवित शिशुलाई जन्म दिनु भएको छ? | ________ | ☐Don't apply/ लागु हुदैंन |
| 29. | How many children are dependent on you? /तपाईमा आश्रित हुने कति जना बालबालिका छन्? | ________ | ☐Don't apply/ लागु हुदैंन |
| 30. | How many adult women (>18 years) are physically present at home? / कति जना बयस्क (१८ वर्ष भन्दा माथिका) महिला भैतिक रुपमा घरमा हुनुहुन्छ? | ________ | ☐Don't apply / लागु हुदैंन |
| 31. | How many female children (<18 years) live in the home? / घरमा कति जना महिला बच्चा (१८ वर्ष भन्दा मुनिका) बस्नु हुन्छ? | ________ | ☐Don't apply/ लागु हुदैंन |

| | | | | |
|---|---|---|---|---|
| 32. | What is your occupation? / तपाईँको पेशा के हो? | ☐Student /विधार्थी<br>☐Farmer /कृषक | ☐Homemaker/ गृहिणी<br>☐Business / व्यापार | ☐Others/ अन्य |
| 33. | What is your husband's occupation? / तपाईँको श्रीमानको पेशा के हो? | ☐Student /विधार्थी<br>☐Farmer /कृषक | ☐Homemaker/ घर व्यवस्थापक<br>☐Business/ व्यापार | ☐Other/ अन्य<br>☐Don't apply/ लागु हुदैंन |
| Skip question "33" if women are single/unmarried/widowed/divorced. / यदि महिलाहरु एकल/अबिवाहित/विधवा/परपाचुके भएका छन् भने प्रश्न "33" को जवाफ आवश्यक छैन। | | | | |

| | | |
|---|---|---|
| 34. | What is the annual income of your family? / तपाईँको परिवारको वार्षिक कुल आय कति हो? | ______________________ (# rupees/ रुपियाँमा) |

| | | | |
|---|---|---|---|
| 35. | Can you read and write in any language? / के तपाईँ कुनै | ☐Literate/ | ☐Illiterate/निरक्षर |

| | | | |
|---|---|---|---|
| | भाषामा लेख्न र पढ्न सक्नुहुन्छ? | साक्षर | |

| | | | |
|---|---|---|---|
| 36. | What is your highest education level? / तपाईंले प्राप्त गर्नु भएको उच्चतम शिक्षा के हो? | ☐No education/ अशिक्षित<br>☐Basic education (1-8)/ आधारभुत शिक्षा (१-८) | ☐Secondary education (9-12)/ माध्यमिक शिक्षा (९-१२)<br>☐ More than secondary (13 and above) / माध्यमिक शिक्षा भन्दा माथी (१३ वा माथि) |

| | | | | |
|---|---|---|---|---|
| 37. | Did anyone in your family (siblings/parents/children's) have **cervical cancer?** के तपाईंको परिवारमा कसैलाई (दिदीबहिनी/अभिभावक/ बालबालिका) लाई पाठेघरको मुखको क्यान्सर भएको छ? | ☐Positive/ सकारात्मक | ☐Negative/ नकारात्मक | ☐Don't know / थाहा छैन |
| 38. | Who had cervical cancer? /कसलाई पाठेघरको मुखको क्यान्सर भएको थियो? | ________________ (relation to participant/ सहभागि सगँको सम्बन्ध) | | |
| | (Skip question no. 38, if there is no family history of cervical cancer)/ यदि परिवारमा कुनै पनि पाठेघरको क्यान्सरको ईतिहास छैन भने प्रश्न 38 को जवाफ आवश्यक छैन। | | | |
| 39. | Who makes decisions about the health matters in your family? /तपाईंको परिवारमा स्वास्थ सगँ सम्बन्धित निर्णय कसले गर्छ? | | | |
| 40. | Who influences your health decisions for yourself? / तपाईंको स्वास्थ सम्बन्धि निर्णयहरुमा कसले प्रभाव पार्छ? | | | |
| 41. | **Where do you seek help at first if you or your family gets sick?** तपाई वा तपाईको परिवारमा कोहि बिरामी हुनुहुदा सहयोगको लागि सिबै भन्दा पहिला काहाँ जानुहुन्छ? | ☐Hospital/Health post/अस्पताल वा स्वास्थ्य चौकि<br>☐Dhami/ Jhakri/ धामी वा झाक्रि | ☐Lama/लामा<br>☐Mata/माता<br>☐Other/अन्य | ☐Prefer not to answer/ उत्तर दिन चाहन्न्<br>☐Don't know/ थाहा छैन् |

अन्तरवार्ता संख्या/Interview ID __________          मिति/Date________________

घरधुरी संख्या/Household ID________________          समय/Time________________

| | | | | |
|---|---|---|---|---|
| 42. | Which religion do you follow? / तपाईँ कुन धर्ममा विश्वास गर्नु हुन्छ? | ☐Hindu/ हिन्दु<br>☐Buddhist/बुध्धिष्ट | ☐Kirat/किरात<br>☐Muslim/ईस्लाम | ☐Christian/ ईसाई<br>☐Other/अन्य |
